# Qualitative and Quantitative Computed Tomography Analyses of Lung Adenocarcinoma for Predicting Spread Through Air Spaces

**DOI:** 10.3390/tomography11070076

**Published:** 2025-06-27

**Authors:** Fumi Kameda, Yoshie Kunihiro, Masahiro Tanabe, Masatoshi Nakashima, Taiga Kobayashi, Toshiki Tanaka, Yoshinobu Hoshii, Katsuyoshi Ito

**Affiliations:** 1 Department of Radiology, Yamaguchi University Graduate School of Medicine, Ube 755-8505, Japan; natsukusa_fumi@yahoo.co.jp (F.K.); m-tanabe@yamaguchi-u.ac.jp (M.T.);; 2 Department of Radiology, Ube Central Hospital, Ube 755-0151, Japan; 3 Department of Surgery and Clinical Science, Division of Chest Surgery, Yamaguchi University Graduate School of Medicine, Ube 755-8505, Japan; 4 Department of Diagnostic Pathology, Yamaguchi University Hospital, Ube 755-8505, Japan

**Keywords:** spread through air spaces, X-ray-computed tomography, lung adenocarcinoma, quantitative analysis, multivariate analysis

## Abstract

Background/Objectives: Spread through air spaces (STAS) is defined as the spread of tumor cells into the parenchymal alveolar space beyond the margins of the main tumor, and it is associated with worse clinical outcomes in resected lung adenocarcinoma. This study aimed to evaluate the preoperative computed tomography (CT) findings of primary lung adenocarcinoma in surgically resected T1 cases and to compare CT findings with and without STAS. Methods: A total of 145 patients were included in this study. The following factors were evaluated on CT images: nodule type (pure ground-glass nodule [GGN], part-solid nodule, or solid nodule), margin (smooth or irregular), the presence of lobulation, spicula, cavity, calcification, central low attenuation, peripheral opacity (well-defined or ill-defined), air bronchogram, satellite lesions, pleural retraction, pulmonary emphysema, and interstitial pneumonia; CT values (maximum, minimum, and mean); volume (tumor and solid component); and diameter (tumor and solid component). CT criteria were compared between the presence and absence of STAS. Results: Lobulation and central low attenuation were significantly more frequent in patients with STAS (*p* < 0.05). The mean CT value, and the volume, rate, and diameter of the solid component were significantly larger in cases with STAS (*p* < 0.05). A multiple logistic regression analysis identified central low attenuation as an indicator of the presence of STAS (*p* < 0.001; odds ratio, 3.993; 95% confidence interval, 1.993–8.001). Conclusions: Quantitative and qualitative analyses are useful for differentiating between the presence and absence of STAS.

## 1. Introduction

Primary lung cancer has high mortality rates worldwide, and non-small-cell lung cancer (NSCLC) accounts for 80% to 85% of all primary lung cancers [1,2]. In evaluations using computed tomography (CT) for lung cancer, the CT classification is widely adopted, especially for adenocarcinoma. Since the eighth lung cancer tumor-node-metastasis (TNM) classification, radiologic staging using CT depends on the overall tumor size and the presence and size of the solid component, which is associated with the invasiveness of the lung adenocarcinoma [3]. Adenocarcinoma in situ (AIS) usually shows a pure ground-glass nodule (GGN), minimally invasive adenocarcinoma (MIA) usually shows a ground-glass predominant nodule with a ≤0.5 cm solid component, and invasive adenocarcinoma (IAC) usually shows a nodule with a >0.5 cm solid component [3]. In addition, the characteristic CT findings of small lung cancer include notch (or lobulation), spicula (or spiculation), pleural retraction, and small cavitation, which consists of small bronchioles or bronchiectasis, and necrosis [4]. It is reported that the CT volumetry of lung nodules is also useful for quantitative analysis [5]. Recently, the CT workstation based on a three-dimensional (3D) analysis system has been widely used, and it enables an artificial intelligence (AI)-driven measurement of the volume of the overall tumor and the volume of the solid and GGN components.

Spread through air spaces (STAS) is defined as the spread of tumor cells into the parenchymal alveolar space beyond the margins of the main tumor, and it was identified in the World Health Organization (WHO) classification in 2015 as one of the modes of lung cancer progression [6,7]. STAS is associated with worse clinical outcomes in resected lung adenocarcinoma and all investigated major histological types of lung cancer [7]. The presence of STAS is strongly associated with overall and recurrence-free survival, and it is an important clinicopathological predictor of recurrence and prognosis [8]. It has been reported that STAS was found in 22.9% of the pathologic stage I NSCLC, and limited resection alone is associated with a risk of recurrence in clinical stage I primary lung adenocarcinoma with STAS positivity [6,9]. However, it is reportedly difficult to diagnose the presence or absence of STAS based on an intraoperative rapid histological diagnosis using frozen pathological specimens [10]. The preoperative prediction of the STAS status based on CT findings would be an effective method.

There have been several reports of STAS and CT imaging findings in lung adenocarcinoma [10,11,12,13]. However, the relationship between qualitative and quantitative CT analyses and the presence of STAS has not been properly investigated.

The purpose of this study was to define CT findings for predicting the presence of STAS. First, we evaluated the preoperative CT findings using qualitative and quantitative analyses of primary lung adenocarcinoma in surgically resected T1 cases. Next, we compared the CT findings between the STAS-positive and the STAS-negative cases. Finally, a statistical analysis was performed to detect the indicator for predicting the presence of STAS.

## 2. Materials and Methods

### 2.1. Patients

**Figure 1:**
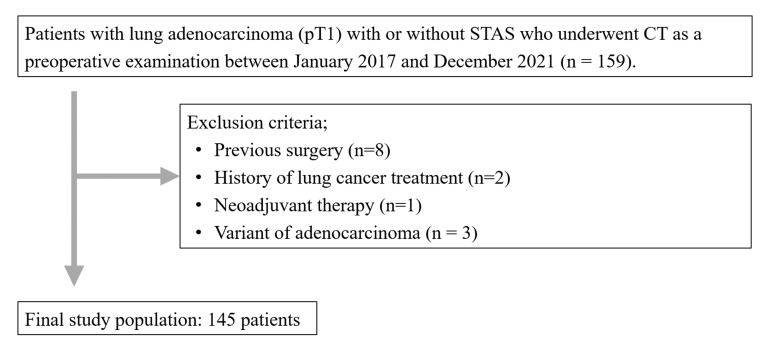
Flowchart of patient selection. Note—STAS, spread through air spaces; CT, computed tomography.

Our institutional review board approved this retrospective study and waived the requirement for informed consent. This study included patients who underwent preoperative CT for lung cancer between January 2017 and December 2021. The inclusion criteria were patients who underwent CT imaging within two months before surgery and who were pathologically diagnosed with pT1 lung adenocarcinoma based on the evaluation of STAS. During the study period, 159 patients met our inclusion criteria. Patients with a history of lung surgery (*n* = 8), lung cancer treatment (*n* = 2), neoadjuvant therapy (*n* = 1), and variants of adenocarcinoma (*n* = 3) were excluded (Figure 1). Thus, a total of 145 patients (male, *n* = 78; female, *n* = 67; mean age: 71.4 ± 9.2 [range: 37–86 years]) were finally enrolled in this study (Table 1). Diagnoses of lung adenocarcinoma included MIA (*n* = 25) and IAC (*n* = 120).

### 2.2. CT Examinations

All chest CT scans were obtained within 2 months before surgery using a 64-row detector CT scanner (SOMATOM FORCE; Siemens Healthineers, Erlangen, Germany) with a slice thickness of 1 mm without a gap. The scans were obtained with the suspended-end inspiration effort in the supine position without intravenous contrast medium injection. The scanning parameters were 100 kVp and automated mA. All image data were interfaced directly with the picture archiving and communication system (PACS) (ShadeQuest; Fujifilm Medical Solutions Corp., Tokyo, Japan), and monitors were used to view the CT image (lung settings: window width [WW], 1500 Hounsfield unit [HU]; window level [WL], −600 HU; mediastinal settings: WW, 255 HU; WL, 50 HU). The reconstructed CT images were qualitatively reviewed by radiologists.

### 2.3. CT Analysis

**Figure 2:**
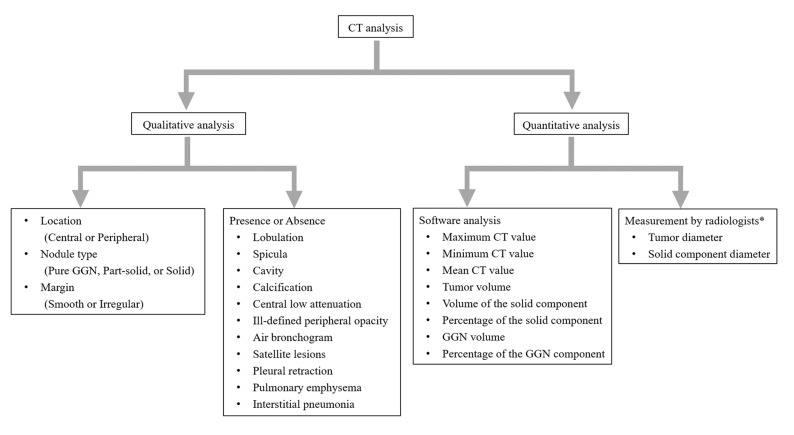
Flowchart of patient selection. Note—CT, computed tomography; GGN, ground-glass nodule; Max, maximum; Min, minimum. * Average data of the measurements by two radiologists are used.

Two thoracic radiologists (Y.N. and F.K., with 1 and 10 years of experience, respectively) independently evaluated the CT images. Discordant results between the two radiologists were resolved by consensus. The following CT features were coded: (a) location (central or peripheral); (b) preoperative biopsy, (c) nodule type (pure GGN, part-solid, or solid nodule); (d) margin (smooth or irregular); (e) lobulation; (f) spicula; (g) cavity; (h) calcification; (i) central low attenuation; (j) peripheral opacity (well-defined or ill-defined); (k) air bronchogram; (l) satellite lesions; (m) pleural retraction; (n) pulmonary emphysema; and (o) interstitial pneumonia. A tumor located entirely within the inner two-thirds of the lung was defined as a central tumor, and a peripheral tumor was defined as being located in the outer third of the lung [8]. Preoperative biopsy results included bronchoalveolar lavage (BAL), transbronchial lung biopsy (TBLB), and CT-guided percutaneous biopsy, and they were recorded as positive or negative for cancer. Pure GGN was defined as a nodule with pure ground-glass attenuation, solid nodule was defined as a nodule with soft tissue attenuation, and part-solid nodule was defined as a nodule with both ground-glass and soft tissue attenuation components [14]. The appearance of the nodule margin was classified as smooth or irregular. Lobulation is the descriptive term referring to a lobule-like and often asymmetric protrusion at the margins of a structure [14]. Spicula (or spiculation) is defined as multiple fine linear strands extending from a nodule into the surrounding lung parenchyma in a stellate manner [14]. A cavity is defined as an abnormal gas within a nodule [14]. Calcification was defined as areas of high attenuation (visually as opaque as bone structures) within a nodule [15]. Central low attenuation was defined as an area of low attenuation within a solid or part-solid lesion using the mediastinal window setting [8]. The appearance of a border line between a nodule and surrounding lung parenchyma was classified into well-defined and ill-defined peripheral opacity. Air bronchogram is the descriptive term for air-filled airways within a nodule [14]. Satellite lesions were defined as smaller nodules located within 2 cm of the primary tumor [8]. Pulmonary emphysema is characterized by enlarged airspaces within the lung parenchyma [14]. The criteria of interstitial pneumonia in this study include the findings of interstitial lung abnormalities; ground-glass opacity and reticular opacity with or without traction bronchiectasis and honeycombing, which affect more than 5% of the upper, middle, or lower lung zone, as demarcated by the levels of the inferior part of the aortic arch and the right inferior pulmonary vein, respectively [14,16]. A quantitative CT analysis of tumors was conducted using the SYNAPSE VINCENT software (v7.0.0003) program (Fujifilm Corp, Tokyo, Japan). One radiologist (F.K.) dragged the tumor and the following quantitative data were automatically analyzed: (a) maximum (max) CT value, (b) minimum (min) CT value, (c) mean CT value, (d) tumor volume, (e) the volume of the solid component (solid volume) and (f) the percentage of the solid component (solid percentage). The radiologist confirmed those results. In addition, the two radiologists measured the diameter of a nodule, including the solid and GGN component, and the diameter of the solid component within a nodule on the axial, sagittal, and coronal section images at first. And then, the longest diameters among them ([g] max tumor diameter and [h] max solid diameter) on any one of the axial, sagittal, and coronal CT images were recorded. The average data between the measurements by the two radiologists were evaluated. Figure 2 shows a flowchart of the CT analysis.

### 2.4. Statistical Analysis

Sex, lymph node (LN) metastasis, tumor location, preoperative biopsy results, and qualitative CT findings were compared between the STAS-positive and STAS-negative groups using the chi-square test or Fisher’s exact test, as appropriate. When the chi-square test detected a significant difference between the groups, adjusted standardized residuals were calculated to identify the groups for which the CT findings contributed to the significant difference. An adjusted standardized residual of >1.96 or <−1.96 was considered indicative of a group with a significantly higher or lower frequency, respectively.

The age and quantitative analysis data of the CT images were compared using the Mann–Whitney U test. A receiver operating characteristic (ROC) curve analysis was used to determine the optimal cutoff values of continuous variables derived from the Youden index, and the area under the curve (AUC) was calculated. The forward selection (likelihood ratio) method was used for the multiple logistic regression analyses to identify significant indicators for differentiating between cases with and without STAS. A multicollinearity diagnosis analysis was utilized to evaluate multicollinearity, and the mean CT value, solid percentage, and max solid diameter were excluded. All other variables, including parametric factors, were included. If independent variables had a variance inflation factor (VIF) < 5, there was no serious multicollinearity among the independent variables. In addition, the nodule type, tumor volume, solid volume, solid percentage, max tumor diameter, and max solid diameter were compared between the groups with and without preoperative biopsy.

Statistical significance was set to *p* < 0.05. Data were analyzed using SPSS (version 27.0, IBM, Armonk, NY, USA).

## 3. Results

### 3.1. Patients’ Characteristics and CT Analysis

**Figure 3:**
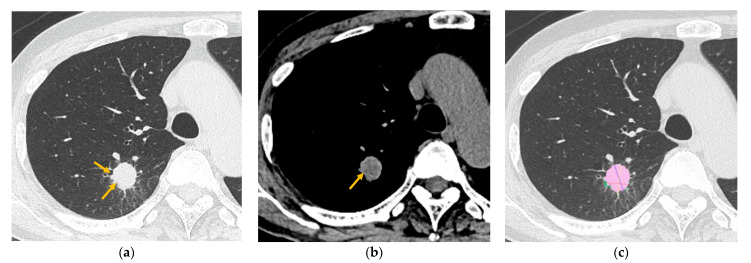
A 70-year-old man with invasive adenocarcinoma (positive for STAS). (**a**) High-resolution computed tomography (HRCT) revealed a solid nodule with lobulation (arrows). The diameters of both the tumor and solid components were 21.0 mm. The rate of the solid component diameter was 100%. (**b**) Central low attenuation was observed (arrow). (**c**) The volume of the tumor and solid component were 2954.7 mm^3^ (pink and green area) and 2954.3 mm^3^ (pink area), respectively. The rate of the solid component volume was 99.986%. Note—STAS, spread through air spaces; HRCT, high-resolution CT.

**Figure 4:**
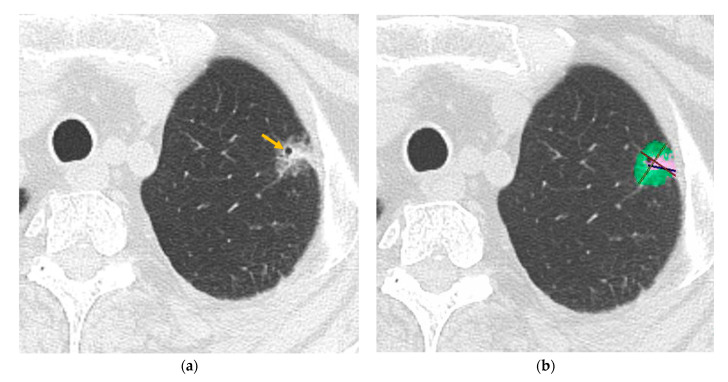
An 80-year-old woman with invasive adenocarcinoma (negative for STAS). (**a**) HRCT shows a part-solid nodule with an air bronchogram (arrow). The diameter of the tumor is 16.6 mm, and the diameter of the solid component is 8.0 mm. The rate of the solid component diameter is 48.2%. (**b**) The volume of the tumor and solid component are 1650.9 mm^3^ (pink and green area) and 325.0 mm^3^ (pink area), respectively. The rate of solid component volume is 19.7%. Note—STAS, spread through air spaces; HRCT, high-resolution CT.

The characteristics of the patients and the CT features according to the STAS status are shown in Table 1. In the patients’ characteristics, age, sex, and the location of the tumor (central or peripheral) were not significantly different between the STAS-positive and STAS-negative groups (*p* > 0.05). LN metastasis (*p* = 0.011) and preoperative biopsy (*p* = 0.041) were significantly more frequent in the STAS-positive group. There was no significant association between carcinoma positivity through biopsy and STAS positivity (*p* = 0.472). In the qualitative CT analysis, lobulation (*p* = 0.004) and central low attenuation (*p* < 0.001) were significantly more frequent in the STAS-positive group (Figure 3), and air bronchograms were significantly more frequent in the STAS-negative group (*p* = 0.005) (Figure 4). The nodule type (pure GGN, part-solid, or solid nodule), the margin of the nodule (smooth or irregular), the spicula, the cavity, calcification, peripheral opacity, satellite lesions, pleural retraction, pulmonary emphysema, and interstitial pneumonia were not significantly different between the STAS-positive and STAS-negative groups (*p* > 0.05). In the quantitative CT analysis, the mean CT value (*p* = 0.003), solid volume (*p* < 0.001), solid percentage (*p* = 0.002), and max solid component diameter (*p* < 0.001) were significantly larger in the STAS-positive group. Tumor volume and the max tumor diameter were not significantly different between the STAS-positive and STAS-negative groups (*p* > 0.05).

### 3.2. ROC Curve Analysis and Multiple Logistic Regression Analyses

**Figure 5:**
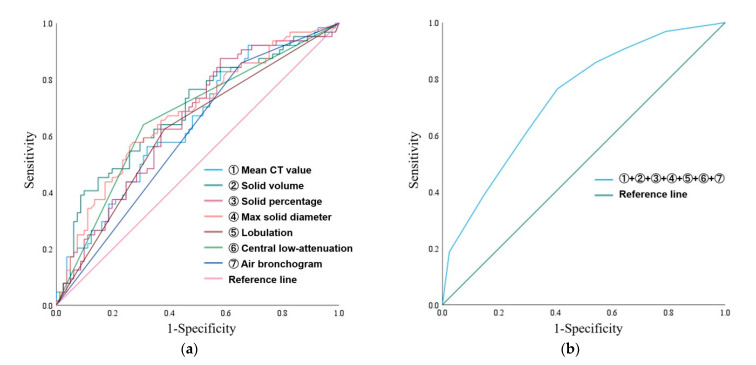
ROC curves. (**a**) ROC curves predicting the presence of STAS based on the mean CT value (light blue line), solid volume (dark green line), solid percentage (dark pink line), max solid diameter (orange line), lobulation (brown line), central low attenuation (light green line), and the absence of an air bronchogram (dark blue line). (**b**) ROC curves predicting the presence of STAS based on the combination of qualitative and quantitative CT findings. Note—ROC, receiver operating characteristic; STAS, spread through air spaces; CT, computed tomography.

For the qualitative and quantitative CT findings, ROC curve analyses for predicting STAS were performed (Table 2 and Figure 5a). The AUC of solid volume was the highest (0.687; 95% confidence interval [CI] 0.599–0.775), and the optimal cutoff was 2055.2 mm^3^ (sensitivity and specificity were 0.406 and 0.901, respectively) (Table 2). The AUC for predicting the presence of STAS was 0.731 (95% CI, 0.651–0.812) when qualitative and quantitative CT findings (mean CT value, solid volume, solid percentage, max solid diameter, the presence of lobulation, central low attenuation, and the absence of air bronchogram) were classified using cutoff values (Figure 5b).

A multiple logistic regression analysis identified central low attenuation as an indicator of the presence of STAS (*p* < 0.001; odds ratio, 3.993; 95% CI, 1.993–8.001).

### 3.3. Comparative Analysis Between the Cases With or Without Preoperative Biopsy

Table 3 shows a comparison of the type, volume, and diameter of the nodules with and without preoperative biopsy. The nodule type was significantly different (*p* = 0.029), and solid nodules were significantly more frequent in the biopsy group. Tumor volume (*p* = 0.009), solid volume (*p* < 0.001), solid percentage (*p* < 0.001), max tumor diameter (*p* = 0.003), and max solid diameter (*p* = 0.001) were significantly larger in the biopsy group.

## 4. Discussion

Our study showed a correlation between quantitative and qualitative CT data and the STAS status. Central low attenuation was a significant indicator of STAS, and the AUC of solid volume was the highest. Additionally, our study showed that combining valuables (mean CT value, solid volume, solid percentage, the max solid diameter, the presence of lobulation, central low attenuation, and the absence of air bronchogram) could improve diagnostic power.

Previous studies reported that STAS is a prognostic marker that is associated with significantly reduced overall and disease-free survival [6,17,18,19,20,21]. STAS is a significant risk factor for recurrence after limited resection [6], and STAS and tumor margins smaller than 1 cm are significant risk factors for local recurrence in early-stage lung cancer after limited resection [22]. Other local-directed therapies, including stereotactic body radiation therapy and percutaneous image-guided ablation, suggest that STAS would be useful for determining adequate ablation margins, which are closely related to local recurrence [23,24].

Our study showed that lobulation and central low attenuation were significantly more frequent in the STAS-positive group, and air bronchograms were significantly more frequent in the STAS-negative group. Lobulation is the descriptive term referring to a lobule-like and often asymmetric protrusion at the margins of a structure; it often reflects different rates of tissue growth or the presence of different tissues within the same structure pathologically, and it typically has smooth margins [14]. Lobulation is often seen in malignant tumors, especially squamous cell carcinoma, poorly differentiated adenocarcinoma, small cell carcinoma, and large cell neuroendocrine carcinoma [4,25,26], though it can be observed in benign tumors. Central low attenuation within the tumor is associated with mucinous features [8]. Air bronchogram is the descriptive term for air-filled airways within lung parenchyma that are partially or completely airless [14]. Though air bronchogram is most frequently seen in pneumonia, it can be found in adenocarcinomas, particularly those with a sub-solid appearance [27]. It is also reported that air bronchogram provided positive prognostic information for recurrence-free survival in patients with pure-solid appearance [27]. It has been reported that the type of nodules, the presence of lobulation, ill-defined opacity, spicula, cavity, central low attenuation, vascular convergence, pleural indentation, and the absence of ground-glass opacity were associated with the STAS status [9,10,16,28]. Our study supported the findings of previous studies, although there was no significant difference in nodule type. Air bronchogram was observed significantly more frequently in STAS-negative tumors in our study. In contrast, in previous studies, the air bronchogram was 45.6–46.7% in STAS-positive tumors [9,10]. Satellite lesions could be associated with macroscopic tumor spread [29,30,31]; however, satellite lesions were not a significant indicator in our study or in another previous study [8].

Previous studies reported that the maximum tumor diameter, the diameter of the solid component, and the percentage of the solid component were associated with the STAS status [10,11,16]. Our study showed that the tumor diameter was not significantly different; however, other data showed significant differences according to the STAS status. In addition, the volume of the solid component was significantly larger in the STAS-positive tumors in our study. In a previous study, STAS-positive tumors were associated with a high CT value [12]. There were no significant differences in the minimum and maximum CT values; however, the mean CT values were significantly higher in the STAS-positive tumors in our study.

Our study showed a significant association between the preoperative biopsy and the STAS status, although there was no significant association between biopsy positivity for carcinoma and STAS positivity. Our study supported that clinicians tend to perform preoperative biopsies for patients who show relatively larger solid components on CT because of the accuracy of the diagnosis. This could have affected the results of the present study. A previous study reported that the performance of a preoperative biopsy was not a significant risk factor for either recurrence or mortality irrespective of STAS positivity [32].

The present study was associated with several limitations. First, the study was retrospective in nature and conducted at a single institution. The relatively small study population limited the power of the statistical analysis. Second, we only evaluated STAS in surgically resected pT1 lung adenocarcinomas and did not examine unresectable lung cancers. Thus, selection bias could occur there. STAS is pathologically defined in surgically resected tumors as the spread of tumor cells into the parenchymal alveolar space beyond the margins of the main tumor. However, future studies should include lung cancer with more advanced tumors than pT1 or with other histological types in order to predict prognosis and the risk of recurrence after surgical resection more properly.

## 5. Conclusions

In conclusion, qualitative and quantitative CT analyses may be useful for predicting STAS. A multiple logistic regression analysis identified central low attenuation as an indicator of the presence of STAS. The solid component was significantly larger in STAS-positive cases. A combination of qualitative and quantitative data with high prediction accuracy could be used additionally.

## Figures and Tables

**Table 1:** Patients’ characteristics and CT features according to STAS status.

	All Patients (*n* = 145)	Positive for STAS (*n* = 64)	Negative for STAS (*n* = 81)	*p* Value
Age	71.4 ± 9.2	71.3 ± 8.7	71.5 ± 9.6	0.658
Sex, *n* (%)				0.848
Male	78 (53.7)	35 (54.7)	43 (53.1)	
Female	67 (46.2)	29 (45.3)	38 (46.9)	
LN metastasis, *n* (%)	11 (7.6)	9 (14.1)	2 (2.5)	0.011
Location, *n* (%)				0.615
Central	40 (27.6)	19 (29.7)	21 (25.9)	
Peripheral	105 (72.4)	45 (70.3)	60 (74.1)	
Preoperative biopsy, *n* (%)	70 (48.3)	37/70 (52.9)	33/70 (47.1)	0.041
Positive for carcinoma	29/70 (41.4)	17/70 (24.3)	12/70 (17.1)	0.472
Negative for carcinoma	41/70 (58.6)	20/70 (28.6)	21/70 (30.0)	
***Qualitative analysis***				
Nodule type, *n* (%)				0.351
Pure GGN	9 (6.2)	2 (3.1)	7 (8.6)	
Part-solid nodule	100 (69.0)	44 (68.8)	56 (69.1)	
Solid nodule	36 (24.8)	18 (28.1)	18 (22.2)	
Margin, *n* (%)				0.647
Smooth	39 (26.9)	16 (25.0)	23 (28.4)	
Irregular	106 (73.1)	48 (75.0)	58 (71.6)	
Lobulation, *n* (%)	71 (49.0)	40 (62.5)	31 (38.3)	0.004
Spicula, *n* (%)	69 (47.6)	35 (54.7)	34 (42.0)	0.128
Cavity, *n* (%)	9 (6.2)	4 (6.3)	5 (6.2)	1.000
Calcification, *n* (%)	7 (4.8)	5 (7.8)	2 (2.5)	0.241
Central low attenuation, *n* (%)	66 (45.5)	41 (64.1)	25 (30.9)	<0.001
Peripheral opacity, *n* (%)				0.327
Well-defined	91 (62.8)	43 (67.2)	48(59.3)	
Ill-defined	54(37.2)	21(32.8)	33(40.7)	
Air bronchogram, *n* (%)	37 (25.5)	9 (14.1)	28 (34.6)	0.005
Satellite lesions, *n* (%)	5 (3.4)	4 (6.3)	1 (1.2)	0.170
Pleural retraction, *n* (%)	66 (45.5)	33 (51.6)	33 (40.7)	0.194
Pulmonary emphysema, *n* (%)	34 (23.4)	18 (28.1)	16 (19.8)	0.237
Interstitial pneumonia, *n* (%)	16 (11.0)	5 (7.8)	11 (13.6)	0.271
***Quantitative analysis***				
Max CT value (HU)	200.1 ± 207.2	216.2 ± 205.3	187.5 ± 209.0	0.168
Min CT value (HU)	−872.2 ± 198.1	−856.8 ± 227.4	−884.3 ± 172.1	0.684
Mean CT value (HU)	−370.0 ± 251.6	−300.1 ± 246.9	−425.2 ± 242.8	0.003
Tumor volume (mm^3^)	3953.4 ± 5606.8	4349.4 ± 5382.7	3640.5 ± 5791.7	0.055
Solid volume (mm^3^)	1588.6 ± 2236.0	2187.2 ± 2506.2	1115.7 ± 1881.5	<0.001
Solid percentage (%)	48.6 ± 33.2	57.8 ± 31.5	41.3 ± 32.9	0.002
Max tumor diameter (mm)	22.6 ± 9.9	23.7 ± 9.0	21.7 ± 10.5	0.063
Max solid diameter (mm)	15.0 ± 9.8	18.1 ± 9.8	12.5 ± 9.1	<0.001

Unless otherwise specified, data are presented as means ± standard deviations. Note—CT, computed tomography; STAS, spread through air spaces; LN, lymph node; GGN, ground-glass nodule; HU, Hounsfield unit; Max, maximum; Min, minimum.

**Table 2:** AUC, optimal cutoff, sensitivity, and specificity for predicting STAS.

	AUC (95% CI)	Optimal Cutoff	Sensitivity	Specificity
***Quantitative analysis***				
Mean CT value (HU)	0.643 (0.553–0.733)	−527.9	0.828	0.420
Solid volume (mm^3^)	0.687 (0.599–0.775)	2055.2	0.406	0.901
Solid percentage (%)	0.651 (0.561–0.740)	24.2	0.875	0.420
Max solid diameter (mm)	0.678 (0.591–0.766)	16.3	0.578	0.728
***Qualitative analysis***				
Lobulation	0.621 (0.529–0.713)	–	–	–
Central low attenuation	0.666 (0.576–0.756)	–	–	–
Air bronchogram	0.603 (0.511–0.694)	–	–	–

Note—AUC, area under the curve; STAS, spread through air spaces; CI, confidence interval; CT, computed tomography; HU, Hounsfield unit; Max, maximum.

**Table 3:** Comparison of type, volume, and diameter of nodules with and without preoperative biopsy.

	Biopsy (*n* = 70)	Non-Biopsy (*n* = 75)	*p* Value
Nodule type, *n* (%)			0.029
Pure GGN	3 (4.3)	6 (8.0)	
Part-solid nodule	43 (61.4)	57 (76.0)	
Solid nodule	24 (34.0) ^†^	12 (16.0) ^‡^	
Tumor volume (mm^3^)	4663.6 ± 6228.2	3290.6 ± 4907.2	0.009
Solid volume (mm^3^)	1942.8 ± 2214.2	1258.1 ± 2220.1	<0.001
Solid percentage (%)	59.2 ± 33.0	38.7 ± 30.4	<0.001
Max tumor diameter (mm)	24.7 ± 9.9	20.7 ± 9.5	0.003
Max solid diameter (mm)	17.5 ± 9.9	12.6 ± 9.2	0.001

Unless otherwise specified, data are presented as means ± standard deviations. ^†^ Significantly higher (adjusted standard residuals > 1.96) in the group. ^‡^ Significantly lower (adjusted standard residuals < −1.96) in the group. Note—GGN, ground-glass nodule; Max, maximum.

## Data Availability

The original contributions presented in this study are included in the article. Further inquiries can be directed to the corresponding author.

## Abbreviations

The following abbreviations are used in this manuscript:

CT	Computed tomography
STAS	Spread through air spaces
GGN	Ground-glass nodule
NSCLC	Non-small-cell lung cancer
TNM	Tumor-node-metastasis
AIS	Adenocarcinoma in situ
MIA	Minimally invasive adenocarcinoma
IAC	Invasive adenocarcinoma
3D	Three-dimensional
AI	Artificial intelligence
WHO	World Health Organization
PACS	Picture archiving and communication system
WL	Window level
WW	Window width
HU	Hounsfield unit
BAL	Bronchoalveolar lavage
TBLB	Transbronchial lung biopsy
Max	Maximum
Min	Minimum
LN	Lymph node
ROC	Receiver operating characteristics
AUC	Area under the curve
CI	Confidence interval
